# Chronic alcohol exposure disrupts top-down control over basal ganglia action selection to produce habits

**DOI:** 10.1038/s41467-017-02615-9

**Published:** 2018-01-15

**Authors:** Rafael Renteria, Emily T. Baltz, Christina M. Gremel

**Affiliations:** 1 Department of Psychology, University of California San Diego, La Jolla, CA 92093 USA; 2 The Neurosciences Graduate Program, University of California San Diego, La Jolla, CA 92093 USA

## Abstract

Addiction involves a predominance of habitual control mediated through action selection processes in dorsal striatum. Research has largely focused on neural mechanisms mediating a proposed progression from ventral to dorsal lateral striatal control in addiction. However, over reliance on habit striatal processes may also arise from reduced cortical input to striatum, thereby disrupting executive control over action selection. Here, we identify novel mechanisms through which chronic intermittent ethanol exposure and withdrawal (CIE) disrupts top-down control over goal-directed action selection processes to produce habits. We find CIE results in decreased excitability of orbital frontal cortex (OFC) excitatory circuits supporting goal-directed control, and, strikingly, selectively reduces OFC output to the direct output pathway in dorsal medial striatum. Increasing the activity of OFC circuits restores goal-directed control in CIE-exposed mice. Our findings show habitual control in alcohol dependence can arise through disrupted communication between top-down, goal-directed processes onto basal ganglia pathways controlling action selection.

## Abstract (web summary)

Drug dependence shifts the balance in action selection away from goal-directed to habitual responding. Here, the authors report that chronic passive exposure to alcohol leads to suppression of orbitofrontal cortex inputs to dorsomedial striatum resulting in downregulation of goal-directed behavior.

## Introduction

A prominent hypothesis in the drug-abuse field is that addiction involves a transition from goal-directed to habitual control over drug-seeking and taking behaviors^1–4^. How this shift in behavioral control emerges and how it contributes to the addiction is not clear. Research has largely focused on a shift in the underlying neural circuits controlling long-term drug-seeking and drug-taking behaviors^4–10^. These studies have demonstrated that habitual drug-seeking depends on dorsal lateral striatum (DLS)^6–8,10–12^. Investigations into the development of habitual control have emphasized a corresponding progression from ventral striatal to dorsal striatal control over drug-related behaviors^5,13,14^. However, a wealth of research on the neurobiology of action control suggests action selection arises through competition between dorsal striatal subregions^15–20^. In particular, both dorsal medial striatum (DMS) and DLS are concurrently capable of controlling the same action, but compete for goal-directed or habitual control over that action, respectively^17,19–21^. Given this, it may be that an over reliance on habits in drug dependence originates from a strengthening of DLS habitual processes, and/or, a disruption to DMS control over goal-directed processes.

Recent reports on addicts have highlighted a hypothesis that habitual control comes to dominate decision-making as a consequence of an impaired goal-directed system^22,23^. Goal-directed processes underlie decision-making^24^, and drug dependence induces long-lasting deficits in goal-directed decision-making processes^22,23,25,26^. For example, previous findings have reported that alcoholics show persistent disruptions in decision-making processes^27,28^ and these disruptions likely contribute to relapse^29^. Dysfunctional decision-making is most likely the result of dependence-induced changes in the structure and function of corresponding corticostriatal circuits^30,31^. However, there is currently no mechanistic understanding as to whether dependence-induced disruption to cortical goal-directed processes directly results in habitual control. Consequently, despite broad interest in the mechanisms through which habits emerge in drug dependence, there is limited information on the contribution of drug dependence-induced changes to cortical function in habit formation.

To directly investigate whether drug dependence itself disrupts goal-directed control to result in a reliance on habitual decision-making, we are using a well-validated and commonly used mouse model, chronic intermittent ethanol exposure and withdrawal (CIE), to produce ethanol dependence^32–37^. In combination with a recently developed instrumental task for food reward, we find that prior CIE produces a long-lasting disruption to goal-directed processes and leave mice reliant on habitual control. We then examined whether CIE induces long-lasting alterations in the function of one corticostriatal circuit known to control goal-directed actions^19,21,38–41^ namely, the orbital frontal cortex (OFC) and its projections into the medial portion of the dorsal striatum (OFC-DMS). We find that prior CIE exposure decreases activity and output of corticostriatal circuits in a projection and cell-type specific manner, with selective reduction in glutamatergic transmission from OFC-DMS projections onto the direct, but not indirect, output pathway of the basal ganglia. Further, we show that increasing the activity of orbital circuits is sufficient to overcome the reliance on habits and restores goal-directed control in CIE-exposed mice. Together, our findings suggest that dependence-induced reliance on habitual control arises in part through disruption of goal-directed processes including top-down cortical communication onto a basal ganglia pathway controlling action selection.

## Results

### Induction of ethanol dependence disrupts decision-making

**Fig. 1:**
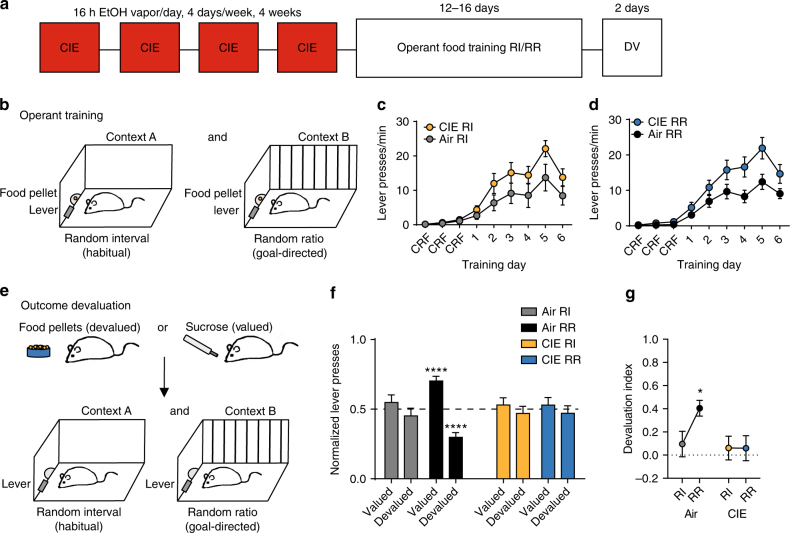
Chronic ethanol exposure and repeated withdrawal biases towards habitual control over actions. **a** Experimental timeline of CIE and the following operant training and subsequent outcome devaluation testing (DV). **b** Mice (3 cohorts, Air *n* = 15, CIE *n* = 19) are trained to press the same lever (left or right) for the same food outcome (food pellet or sucrose) in two distinct contexts under RI or RR schedules of reinforcement. **c**, **d** Response rate of lever pressing during acquisition under RI (**c**) or RR (**d**) schedules. **e** Schematic of the outcome devaluation procedures. On the devalued day, mice receive 1 h free access to the outcome previously produced by lever pressing, immediately followed by a 5 min extinction test in each context. To control for effects of general satiety on responding, on the valued day mice receive 1 h free access to the remaining outcome, immediately followed by a 5 min extinction test in each context. **f** Normalized lever presses showing the distribution of lever pressing between the valued and devalued day in each training context. **g** Devaluation index (see Methods) for each group in the previously trained RI and RR contexts. Data are represented as mean ± SEM. ****p < 0.001, *p < 0.05 reflect one-sample *t* tests against 0.5 and 0, respectively

To investigate whether prior drug dependence results in a long-lasting disruption to decision-making processes, we utilized a well-validated CIE model to induce ethanol dependence in mice^32–37^. Mice were exposed to periods of CIE or air (Air) vapor and subsequent withdrawal over a period of four weeks (Fig. 1a, three vapor cohorts, Air *n* = 15, CIE *n* = 19). Mice were placed in inhalation chambers and exposed to ethanol or air vapor for 16 h per day, 4 days per week. We did not give a loading dose of ethanol or a pretreatment of pyrazole^33^ to avoid confounding effects of stress that can bias reliance on habitual control^42^, as well as to avoid pyrazole’s broad effects on neural activity including actions at the *N*-methyl-d-aspartate (NMDA) receptor^43^. Even without pretreatment, our procedure produced mean blood ethanol concentrations of 34.7 ± 2.0 mM, similar to what has previously been reported^34,44^. After 72 h of the last CIE exposure, mice were food restricted to achieve 90% of their baseline weight for 2 days prior to instrumental lever press training for food pellets or sucrose.

Decision-making recruits parallel action strategies: goal-directed actions and habitual actions^24^. If ethanol dependence does induce long-lasting changes to decision-making processes, it may be apparent in the disrupted use of goal-directed actions or a bias towards reliance on habits. We utilized an instrumental task we recently developed, where on the same day, the same mouse will shift between goal-directed and habitual control over food responding^19,21^. In brief, mice were trained in two distinct contexts to press a lever in the same location for the same food outcome (food pellet or 20% sucrose). To predispose the use of habitual vs. goal-directed action control, mice were trained to lever press under random interval (RI) and random ratio (RR) schedules of reinforcement, respectively (Fig. 1b)^45–47^. Trained under these schedules, Air mice and CIE mice acquired lever press behavior for food (Fig. 1c, d; Supplementary Fig. 1). Although visually, it appeared that CIE exposure increased response rate, a three-way repeated measures ANOVA (context  × CIE exposure × training day) on response rate during training showed a main effect of training day (*F*_(8,112)_ = 30.61, *p* < 0.001), but no main effect of CIE exposure or interaction (*F*s’<1.31). This suggests that while CIE exposure may have led to slightly increased response rates, all mice increased lever pressing in a similar manner across training.

To assess whether an action is goal-directed or habitual, we examined the sensitivity of lever pressing to changes in expected outcome value using outcome devaluation procedures. After 15 to 21 days following the last vapor exposure and lever press training, we subjected Air and CIE mice to sensory-specific satiation of the food outcome (food pellet or sucrose) previously produced by lever pressing (devalued state), or a control outcome (the remaining outcome) mice had previously experienced in their home-cage (valued state) (Fig. 1e). Each prefeeding period was followed by a brief 5-min test in each of the trained contexts, where we measured the number of non-reinforced lever presses made. A significant reduction in lever pressing in the devalued state compared to valued state is indicative of goal-directed control, while similar pressing between states reflects habitual control^48^.

Air mice readily shifted between using a goal-directed strategy in the previously RR trained context and control by more habitual processes in the previously RI trained context, while CIE showed a noted lack of goal-directed control in RI and RR training contexts (Fig. 1f-g; Supplementary Fig. 1e). Since differences in response rates during acquisition and testing were observed within a group, as well as between Air and CIE exposure groups (Supplementary Fig. 1 and 3), lever presses were normalized to total presses made in each context during testing. This allows us to examine CIE effects on decision-making in the absence of differences in response rates. A three-way ANOVA on normalized lever pressing showed a significant three-way interaction (devaluation state × context × CIE exposure: *F*_(1, 32)_ = 10.83, *p* = 0.002), a significant two-way interaction of devaluation state × context (*F*_(1,32)_ = 10.57, *p* = 0.003) and a main effect of devaluation state (*F*_(1,32)_ = 5.20, *p* = 0.03), but no other two-way interactions or main effects (*F*s < 2.00). This suggests that CIE mice and Air mice show different sensitivity to outcome devaluation testing across RI and RR training contexts.

To examine whether Air and CIE mice differentially distributed lever pressing across valued and devalued days, we performed one-sample *t* tests performed against 0.5 (equal lever pressing on valued and devalued days) on normalized lever press data. Air mice differentially distributed lever presses between valued and devalued days in the RR training context (*t*_14_ = 5.95, *p* < 0.0001) but not in the RI context (*t*_14_ = 0.86, *p* = 0.40) (Fig. 1e). In striking contrast to Air mice, CIE mice did not differentially distribute lever pressing between valuation states in either RI or RR trained contexts (*t*s < 0.60) (Fig. 1f). These findings confirm that Air mice reduced responding following outcome devaluation only in the RR context but not RI context, and that CIE exposure resulted in lever pressing insensitive to outcome devaluation in either training context.

We then used a devaluation index to assess whether an individual mouse shifted the degree to which lever pressing was goal-directed between contexts (Methods). We found that CIE exposure disrupts the within-subject shift in goal-directed control. We performed repeated measures ANOVA (context × CIE exposure) and found a significant interaction (*F*_(1,32)_ = 10.83, *p* = 0.002) and a main effect of context (*F*_(1,32)_ = 10.57, *p* = 0.003), but no effect of CIE exposure (*F* < 1.95). Although Air mice showed an increase in goal-directedness in the RR context compared to the RI context (Bonferroni-corrected *p* < 0.001), CIE mice showed similar levels of goal directedness between contexts (*p* > 0.1) (Fig. 1g). One sample *t* tests performed against a hypothetical 0 devaluation index (equal pressing between valued and devalued states) confirmed significant goal-directed control in Air mice only in the RR context (*t*_14_ = 5.95, *p* < 0.001), but not in the RI context (RI *t* = 0.86) or in CIE mice in either RI and RR contexts (*t*s < 0.60).

The lack of goal-directedness in CIE mice cannot be attributed to dependence-induced changes in outcome palatability or sensitivity to devaluation. A subset of Air mice and CIE mice underwent a post-test free feeding assay immediately following outcome devaluation testing. Air and CIE mice consumed similar amounts in prefeeding devaluation procedures as well as in post-test free feeding procedures (Supplementary Fig. 1d, g). Further, correlational analyses performed between the response rate during training and responding during testing suggest that the increased response rate observed in CIE mice did not contribute to the differences in the magnitude of subsequent goal-directed control (Supplementary Fig. 1h). Instead, the present findings suggest that prior CIE exposure results in a long-lasting deficit in decision-making processes, as reflected in the disruption of goal-directed control examined ~3 weeks after the last exposure to ethanol.

### CIE exposure selectively alters orbitostriatal circuits

CIE exposure resulted in long-lasting changes to decision-making processes, suggesting that ethanol dependence-induced changes in neural circuits controlling goal-directed and/or habitual actions. For example, CIE exposure may disrupt neural circuits supporting goal-directed control, or enhance control of neural circuits modulating habits. Previous research has shown long-term changes in the cortical activation of abstinent alcoholics^30^. In particular, hypoactivation of OFC circuits correlated with impaired reward choice behavior in abstinence. Abstinent alcoholics were found to have an immediate reward bias, with BOLD signal in lateral OFC correlated to delayed reward choice^27^. More recently, OFC activity has been shown to support goal-directed action control across species^19,21,38–40,49^, and is an important regulator of the shift between goal-directed and habitual control. We recently showed that increases in excitatory transmission at OFC terminals in dorsal striatum (OFC-DMS) drive goal-directed control, with habitual control emerging from the attenuation of OFC-DMS transmission^21^.

**Fig. 2:**
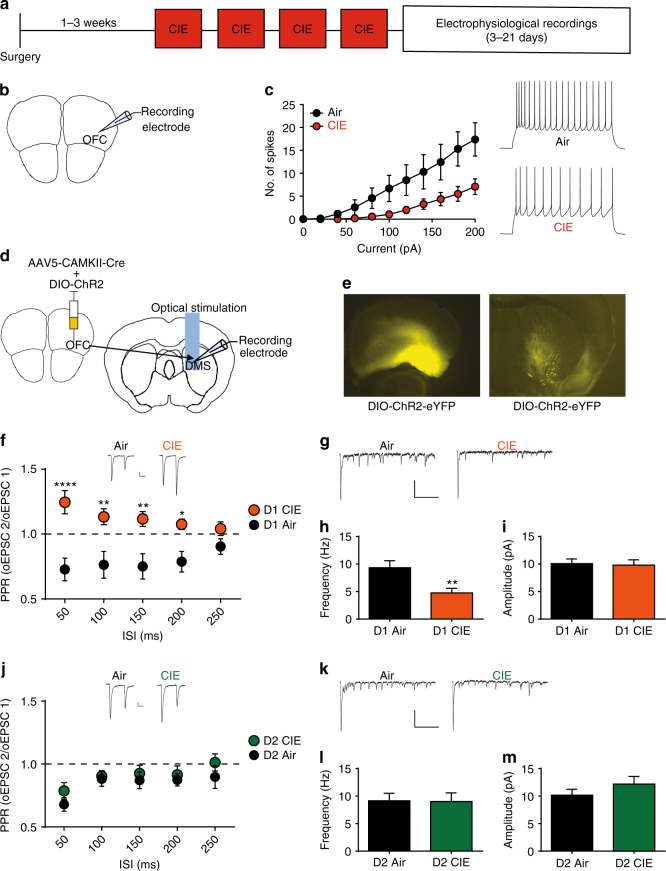
CIE induces long-lasting disruptions to orbitostriatal circuits. **a** Experimental timeline used for electrophysiological recordings. Mice were given viral injections and allowed 2–4 weeks to recover before exposure to the CIE procedure. **b** Schematic of OFC recording site. **c** The number of spikes plotted against current injected (left) and representative traces of action potential firing at 200 pA (right) (3 cohorts, Air *n* = 8, CIE *n* = 11). **d** Schematic of OFC injection site and DMS recording site for optically induced currents. **e** Cre-dependent ChR2-YFP expression at the OFC injection site (left) and OFC terminals in the DMS (right). **f** Paired pulse ratio (PPR) of optically induced currents of OFC input to D1 SPNs. Scale bars represent 25 ms (horizontal) and 50 pA (vertical) (3 cohorts, Air *n* = 7, CIE *n* = 15). **g** Representative current traces of asynchronous release to D1 SPNS recorded in 2 mM Sr^2+^. Scale bars represent 250 ms (horizontal) and 50 pA (vertical). **h** Average frequency of asynchronous release to D1 SPNs (Air *n* = 8, CIE *n* = 12). **i** Average amplitude of asynchronous release to D1 SPNs. **j** PPR of optically induced currents of OFC input to D2 SPNs (3 cohorts, Air *n* = 7, CIE *n* = 9). **k** Representative current traces of asynchronous release to D2 SPNs recorded in 2 mM Sr^2+^. **l** Average frequency of asynchronous release to D2 SPNs (Air *n* = 7, CIE *n* = 7). **m** Average amplitude of asynchronous release to D2 SPNs. Data points and bar graphs represent the average ± SEM. ****p < 0.0001, **p < 0.01, *p < 0.05

Given the importance of OFC-DMS function for goal-directed control over actions, we hypothesized that ethanol dependence alters OFC function through changes in synaptic transmission onto DMS. Mice were exposed to CIE procedures and ex vivo whole-cell electrophysiological recordings were conducted 3–21 days after the last vapor exposure, corresponding to the time frame of acquisition and devaluation testing (Fig. 2a). First, we examined whether dependence alters intrinsic properties of OFC projection neurons. We observed a decrease in excitability of OFC projection neurons following CIE procedures (repeated measures ANOVA: interaction (CIE exposure × current) = *F*_(10, 170)_ = 5.23, *p* < 0.0001; main effect of current = *F*_(10, 170)_ = 27.82, *p* < 0.0001; main effect of CIE exposure = *F*_(1, 17)_ = 5.81, *p* = 0.03) (Fig. 2b, c, Supplementary Table 1; 3 vapor cohorts, Air *n* = 8, CIE *n* = 11) that was present across the 3–21-day range of testing (Supplementary Fig. 2b). In addition, we found that resting membrane potentials were hyperpolarized in CIE-exposed mice compared to Air controls (Supplementary Table 1). This suggests that ethanol dependence induces a long-lasting reduction in the excitability of OFC projection neurons that is present even after a significant period of abstinence.

We next examined whether ethanol dependence would result in changes to OFC-DMS transmission. OFC projection neurons synapse onto spiny projection neurons (SPNs) of both major basal ganglia output pathways in the DMS in similar proportions^50^; SPNs of the direct pathway that express the dopamine-type 1 receptor (D1 SPNs) and SPNs of the indirect pathway that express dopamine-type 2 receptor (D2 SPNs)^51^. Direct and indirect basal ganglia pathways are thought to coordinate activity to support action selection and performance^52^. We hypothesized that OFC-DMS transmission onto direct and indirect pathways may be altered by ethanol dependence. To investigate OFC-DMS circuits in a projection and cell-type-specific manner, we utilized a viral approach in transgenic mice to selectively examine OFC transmission onto D1 or D2 SPNs. To target direct and indirect pathway SPNs, we utilized multiple transgenic lines to ensure the reproducibility of our findings. B6.FVB(Cg)-Tg(Drd1-cre)EY266Gsat/Mmucd (D1-Cre) and B6.Cg-Tg(Drd1a-tdTomato)6Calak/J (D1-tdTomato) transgenic mice were used to target the direct pathway (D1 SPNs), while B6.FVB(Cg)-Tg(Adora2a-cre)KG139Gsat/Mmucd (A2A-Cre) and non-labeled SPNs from D1-tdTomato transgenic lines were used to label the indirect pathway (D2 SPNs). No differences were observed between transgenic lines so results were combined. All mice were injected with AAV5-CamKIIa-GFP-Cre and a Cre-dependent channel rhodopsin (AAV5-Ef1a-DIO-ChR2-eYFP) (UNC viral vector core) in the OFC to limit channel rhodopsin expression to CamKIIa expressing neurons (Fig. 2d, e)^19,21^. D1-Cre and A2a-Cre mice were also injected with AAV5-hSyn-DIO-mCherry targeted to the DMS to label SPN populations. After 1 to 3 weeks of the surgery, mice underwent CIE procedures (Fig. 2a). Following acute withdrawal after the last vapor exposure (3–21 days), whole-cell patch-clamp recordings of identified D1 or D2 SPNs were made and transmission in response to light activation of OFC terminals was examined.

We first used paired pulse ratio (PPR) to examine whether CIE procedures altered the probability of neurotransmitter release from OFC terminals onto D1 SPNs. In Air mice, we found a high probability of neurotransmitter release at the OFC input onto D1 SPNs, as indicated by a paired pulse depression (PPD) (Fig. 2f; Supplementary Fig. 2c). In stark contrast, recordings made from CIE mice showed significant paired pulse facilitation (PPF), revealing a decrease in probability of neurotransmitter release from OFC terminals onto D1 SPNs. A direct comparison between Air and CIE mice using a two-way ANOVA (CIE exposure × interstimulus interval (ISI)) showed a significant interaction (*F*_(4, 68)_ = 3.53, *p* = 0.01), and main effect of CIE exposure (*F*_(1, 17)_ = 16.96, *p* = 0.0007) (Bonferroni-corrected, ****p < 0.0001 vs. Air; **p < 0.0001 vs. Air; *p < 0.05 vs. Air) (Fig. 2f, three vapor cohorts, Air *n* = 7, CIE *n* = 15). To further investigate the observed decrease in neurotransmitter release at OFC-DMS terminals, we replaced Ca^2+^ with strontium (Sr^2+^) in the recording solution and optically stimulated the OFC input. The use of Sr^2+^ in the recording solution has previously been used to examine asynchronous release in an input specific manner, with a decrease in release reflecting a decrease in release probability^53–55^. In CIE mice, we observed a significant decrease in frequency of asynchronous release onto D1 SPNs compared to that observed in Air mice (Student’s *t* test, *t*_18_ = 3.13, *p* < 0.01) with no change in amplitude (Student’s *t* test, *t*_18_ = 0.20, *p* = 0.84) (Fig. 2g–i, Air, *n* = 8, CIE, *n* = 12). This selective decrease in asynchronous release onto the direct pathway was also apparent across the full range of testing (Supplementary Fig. 2d), suggesting a long-lasting decrease in OFC transmission selectively onto the direct pathway.

We next examined whether the induction of ethanol dependence would alter OFC input onto the indirect pathway. Similar to OFC transmission on to D1 SPNs in Air mice, we observed PPD of OFC transmission onto D2 SPNs in Air control mice. However, the PPD of OFC transmission onto D2 SPNs was still present in CIE mice (two-way ANOVA of CIE exposure × ISI: interaction and main effects *F*s’ < 1.0) (Fig. 2j; three vapor cohorts, Air *n* = 7, CIE *n* = 9). When we examined asynchronous release at OFC terminals onto D2 SPNs, we found no differences between Air and CIE mice in the presence of Sr^2+^ (frequency: Student’s *t* test, *p* = 0.96; amplitude: Student’s *t* test, *p* = 0.26) (Fig. 2k–m, Air, *n* = 7, CIE, *n* = 7). Together these results show that prior ethanol dependence-induced long-lasting decreases in OFC neurotransmitter release into DMS in a cell-type-specific manner, selectively affecting transmission onto the direct but not indirect output pathway of the basal ganglia.

**Fig. 3:**
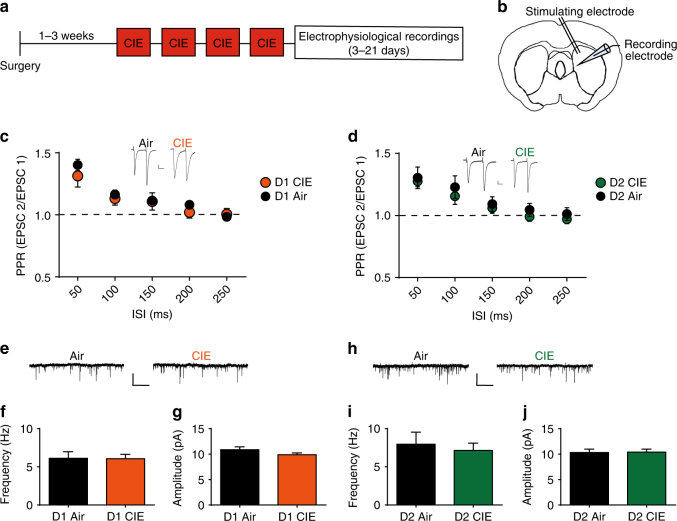
CIE does not alter all excitatory input into striatal circuits. **a** Experimental timeline for cohorts of mice used for electrophysiological recordings. Mice were injected with AAV ChR2 in the OFC and allowed 2–4 weeks to recover before exposure to the CIE procedure. Data collected in which currents were evoked electrically were done in the same brain slices used for optically evoked currents. **b** Schematic of DMS recording site and placement of stimulating electrode within striatum. **c**, **d** Paired pulse ratio (PPR) of electrically induced currents onto D1 SPNs (Air *n* = 6, CIE *n* = 6) (**c**) and D2 SPNS (Air *n* = 7, CIE *n* = 11) (**d**) in Air or CIE-exposed mice. Scale bars represent 25 ms (horizontal) and 50 pA (vertical). **e** Representative traces of spontaneous EPSCs (sEPSCs) in D1 SPNs in Air and CIE mice. Scale bars represent 1 s (horizontal) and 20 pA (vertical). **f**, **d** Frequency (**f**) and amplitude (**g**) of sEPSCs in D1 SPNs from Air and CIE mice. **h** Representative traces of spontaneous EPSCs (sEPSCs) in D2 SPNs in Air and CIE mice. **i**, **j** Frequency (**i**) and amplitude (**j**) of sEPSCs in D2 SPNs from Air and CIE mice. Data points and bar graphs represent the average ± SEM

In addition, the decrease in neurotransmitter release was at least partially selective to the OFC input. When we used electrical stimulation to examine all excitatory input onto either D1 or D2 SPNs (Fig. 3a, b), we found no differences in PPR (no interactions (*p*s > 0.05), D1 SPNs main effect of ISI: *F*_(4, 40)_ = 37.69, *p* < 0.0001; D2 SPNs main effect of ISI: *F*_(4, 40)_ = 16.84, *p* < 0.0001) (Fig. 3c, d). Furthermore, we found no differences between Air and CIE mice in spontaneous EPSC (sEPSC) frequency (*p*s > 0.05) or amplitude in either D1 SPNs (Fig. 3e–g) or D2 SPNs (Fig. 3h–j) (*p*s > 0.05), again suggesting a disruption at least partially selective to OFC-DMS input. Together, our findings suggest that chronic ethanol dependence induces long-lasting decreases in the excitability and output of a pathway known to control goal-directed actions^19,21^, onto a pathway known to support action selection and performance^16,52^. Intriguingly, these changes are mediated through selective changes in OFC transmission onto the direct pathway.

### OFC activation restores goal-directed control following CIE

**Fig. 4:**
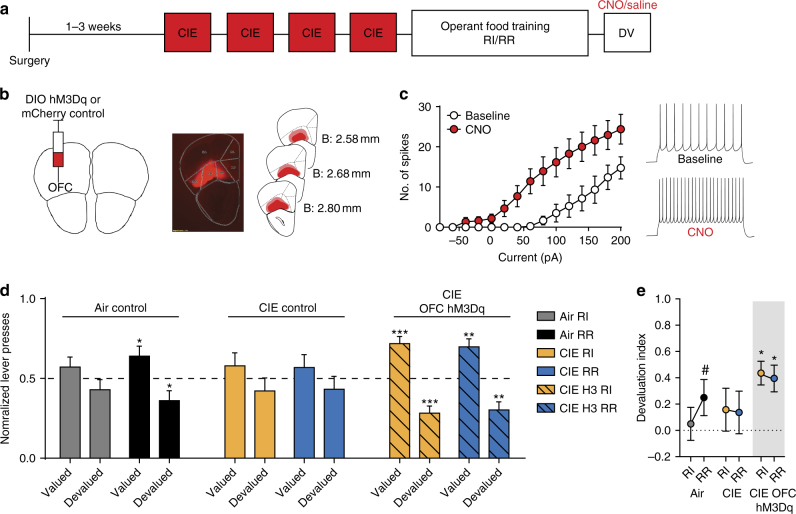
Activation of OFC neurons restores control by goal-directed processes. **a** Experimental outline. Mice were injected with an activating DREADD (hM3Dq) in the OFC. After recovery, mice underwent CIE procedures followed by operant training and outcome devaluation testing. CNO was administered to hM3Dq expressing CIE-exposed mice (CIE H3), control CIE-exposed mice (CIE), and Air-exposed mice (Air) 30 min prior to prefeeding on both valued and devalued testing days. **b** Schematic of OFC injection site with hM3D-mCherry (left) and subsequent expression of mCherry in the OFC (middle). Schematic of maximum (pink) and minimum (red) boundaries of viral spread in the OFC (right). **c** The number of spikes plotted against the current injected (left). Representative traces of action potential firing at 200 pA (right) (*n* = 6 cells). **d** Normalized lever presses for each group (three cohorts, Air *n* = 16, CIE control *n* = 14, CIE H3 *n* = 19) showing the distribution of lever presses between valued and devalued days in random interval (RI) and random ratio (RR) trained contexts. **e** Devaluation index for each group in the previously trained RI and RR contexts. Data are represented as mean ± SEM. **p < 0.01 and ***p < 0.001 reflect one-sample *t* tests against 0.5 and *p < 0.05 and #*p* = 0.09 reflect one-sample *t* tests against 0

While our ex vivo results suggest that the deficits in goal-directed behavior may be in part due to reduced OFC excitability and synaptic transmission into DMS, we do not know whether the observed changes directly biased decision-making. To examine this, we took a chemogenetic approach^56^ to selectively increase OFC projection neuron activity in CIE mice during outcome devaluation testing (Fig. 4a, b). We injected an activating DREADD (AAV5-hSyn-DIO-hM3Dq-mCherry) into the OFC of B6.129S2-Emx1tm1(cre)Krj/J (Emx1-Cre) mice, thereby restricting expression to OFC projection neurons. A subset of Air and CIE mice were injected with AAV5-hSyn-DIO-mCherry (DIO-mCherry) to control for any effects of surgery, AAV infection, and CNO administration. Post-recovery, mice were subjected to CIE exposure procedures, followed by instrumental training and outcome devaluation testing (Fig. 4a, 3 vapor cohorts, groups: Air *n* = 16, CIE control *n* = 14, CIE H3 *n* = 19). To confirm the function of our manipulation, we conducted whole-cell current clamp recordings in identified OFC projection neurons expressing mCherry from infusions of AAV5-hSyn-DIO-hM3Dq-mCherry (Fig. 4b). Bath application of CNO (10 µM) resulted in a significant increase in excitability (two-way repeated measures ANOVA (current × CNO), interaction: *F*_(14, 70)_ = 7.52, *p* < 0.0001; main effect of CNO: *F*_(1, 5)_ = 13.95, *p* = 0.01) (Fig. 4c, *n* = 6).

Following CIE procedures, all mice underwent instrumental lever press training for food (Supplementary Fig. 3). Prior to outcome devaluation testing, all mice were given pretreatments of saline or CNO (1 mg/kg, 10 ml/kg). We used virus and drug treatment controls in each Air and CIE control group and did not see differences between controls injected with saline or CNO; therefore, we collapsed across controls for ease of presentation. While Air mice showed more goal directedness in the RR vs. RI context (albeit to lesser degree), and CIE mice showed little sensitivity to outcome devaluation and were habitual in both contexts, CIE H3 mice showed goal-directed control in both RI and RR contexts. This was supported by a three-way repeated measures ANOVA performed on normalized lever presses (devaluation state × context × group) that did not show a significant three-way interaction (*F*_(2,46)_ = 1.29, *p* = 0.28), but did show a significant two-way interaction between context and group (*F*_(2,46)_ > 15.0, *p* < 0.001), suggesting that Air, CIE, and CIE H3 groups showed different patterns of lever pressing in RI and RR training contexts (Fig. 4d, Supplementary Fig. 3f). A main effect of context (*F*_(1,46)_ > 15, *p* < 0.001) and devaluation state (*F*_(1,46)_ = 14.24, p < 0.001) was also observed, showing that on average, lever pressing differed between RI and RR training contexts and as well as between valued and devalued states.

The finding of different patterns of lever pressing between groups was further supported by one-sample *t* tests against 0.5 conducted on normalized lever pressing. While Air mice differentially distributed lever pressing only in the RR context (albeit slightly) (*t*_15_ = 2.24, *p* < 0.05) and not in the RI context (*t*_15_ = 1.12, *p* = 0.28), CIE mice did not differentially distribute lever pressing between valuation states in either context (one-sample *t* test, RI: *t*_13_ = 0.96; RR: *t*_13_ = 0.84). CNO administration to CIE mice expressing the activating DREADD in OFC projection neurons (CIE H3) restored goal-directed control in the RR context (one-sample *t* test, *t*_18_ = 3.90, *p* < 0.01) and resulted in goal-directed control in the RI context (one-sample *t* test, *t*_18_ = 4.85, *p* < 0.001) (Fig. 4d).

This pattern of H3 activation in CIE producing goal-directed control in otherwise habitual mice was again observed when we examined the within-subject shift in goal directedness using the devaluation index (Fig. 4e). CIE H3 mice displayed greater goal-directed control with index values closer to 1 in both contexts, while Air mice showed slight goal-directed control in the RR, but not RI context. CIE mice showed little goal-directed control in either context (values closer to 0). These findings were supported by a two-way repeated measures ANOVA that examined whether mice differentially shifted action control between contexts. While there was not a significant interaction (context × group; *F* < 0.6) or main effect of context (*F* < 0.66), it did reveal a main effect of group (*F*_(2,44)_ = 3.21, *p* = 0.04), confirming that in general, the groups showed different magnitudes of goal-directedness. To examine if mice showed significant goal-directed control (closer to 1) vs. habitual control (closer to 0), we performed one sample *t* tests performed against a devaluation index of 0. While Air mice tended to show less goal-directed control overall, there was a trend toward greater goal-directedness in the RR context (*t*_13_ = 1.82, *p* = 0.09) but not in the RI context (*t*_13_ = 0.39, *p* = 0.70). CIE mice did not show significant goal-directedness in either training context and did not differ from zero in either context (*t*s < 0.96, *p*s > 0.3). In contrast, H3 activation in CIE H3 mice led to significant goal-directedness in the RI (*t*_18_ = 4.85, *p* < 0.001) and RR (*t*_18_ = 3.91, *p* < 0.01) contexts. Our results show increasing OFC projection neuron activity alone is sufficient to restore goal-directed control in ethanol dependent mice. This suggests that changes in OFC-DMS activity do contribute to the disruption in goal-directed decision-making. Importantly, increasing OFC activity was sufficient to overcome any other neural circuit change that may be predisposing habitual control following the induction of dependence.

## Discussion

The data presented in this study uncover neural mechanisms through which chronic ethanol exposure and withdrawal disrupts decision-making and results in a predominance of habitual control. Our results show that long-lasting dependence-induced changes in the function of goal-directed circuits contributes strongly to the reliance on habitual control. By targeting our investigation in a cell-type and projection-specific manner, we identified dependence-induced changes in OFC excitability and OFC transmission onto the direct, but not indirect, output pathway of the basal ganglia known to contribute to goal-directed control.

To avoid the confound of extended training on the emergence of habits present in long-term drug self-administration and oral consumption experiments, we employed the widely used and well-validated CIE procedure to model ethanol dependence. Therefore, we were able to examine the direct effect of chronic ethanol exposure and withdrawal on the subsequent ability to use decision-making circuits. We followed CIE procedures with a recently developed instrumental training paradigm where we can examine the shift between goal-directed and habitual action control in the same mouse, on the same day^19,21^. In multiple experiments, each with replicating cohorts, we found that CIE exposure disrupts the ability to shift and use goal-directed action strategies indexed by lever pressing behaviors (Figs. 1 and 4). This is in line with previous observations that drug-exposure itself may bias habitual control^57–59^. Thus it appears that the direct effects of CIE exposure and repeated withdrawal are sufficient to disrupt decision-making processes, in the absence of extended drug-self-administration training.

By examining changes ex vivo in corticostriatal circuits post-dependence during the time course that corresponds to action learning and performance, we identified long-lasting dependence-induced changes in OFC excitability and OFC-DMS transmission (Fig. 2). Our data suggest these changes do contribute to the loss of goal-directed control, since increasing OFC activity via activation of G_q_-coupled hM3D receptors in OFC projection neurons in CIE mice specifically during outcome devaluation testing restored goal-directed control (Fig. 4). However, we did not dissociate effects of OFC activation on contributions from changes in excitability from changes in transmission. Further, our data does not address the disrupted aspect of goal-directed control such as updating value vs. using value, or effects on contingency control. It is highly unlikely that OFC and OFC-DMS circuits are the sole decision-making circuit altered following dependence^3^. Further investigation is needed into potential alterations in corticostriatal circuits and their contribution to the reliance on habits.

The induction of ethanol dependence resulted in fairly long-lasting changes in OFC circuit function. We observed a decrease in OFC excitability and synaptic transmission that persisted for up to 21 days after the last vapor exposure while the devaluation test was conducted 15–21 days in withdrawal (Fig. 2; Supplementary Fig. 2). This differs from a previous report that CIE exposure results in a shorter term hyperexcitability of OFC neurons observed 3–10 days after exposure^60^. Here, we used the same CIE procedure and obtained similar BECs but did not use pyrazole, suggesting that the discrepancy in OFC excitability may be due to off-target actions of pyrazole^43^. However, our findings are in line with a recent study on long-term, heavy drinking macaques that found a similar decrease in OFC neuron excitability^61^. In the present study, the reduced OFC excitability following dependence was accompanied by a decrease in OFC synaptic transmission selectively onto the direct, but not indirect, output pathway of the basal ganglia. The observed changes in OFC function and transmission likely contributed to both the development and reliance on habitual control over actions, as these long-term changes were still present at a time point that corresponded to devaluation testing.

Limited work has been done examining effects of chronic ethanol consumption or exposure on glutamatergic activity in dorsal striatum^3^. Here we found a selective reduction in the probability of OFC transmission onto D1 SPNs of the direct pathway following the induction of ethanol dependence in the medial portion of the dorsal striatum (Fig. 2). When we probed transmission onto D1 or D2 SPNs from all excitatory inputs via intra-striatal electrical stimulation, we did not find an effect of dependence, suggesting that dependence selectively changes OFC-mediated inputs to D1 SPNs. (Fig. 3). Our finding of intact transmission at OFC-D2 SPNs suggests that indirect pathway function alone is insufficient for DMS-dependent goal-directed behavior. Previous reports have implicated functional changes in D1 SPNs of the upper DMS following chronic alcohol consumption^62^, with a recent report of selective potentiation of NMDA-mediated synaptic activity in these D1 SPNs during acute withdrawal from chronic alcohol consumption^63^. In contrast, recordings made from SPNs in the DLS (putamen) of very long-term ethanol drinking non-human primates 28 days into abstinence, did find increases in the frequency, but not amplitude of glutamatergic mEPSCs^64^. Given these findings, the consistency of chronic alcohol consumption and exposure effects on glutamatergic activity across striatal subregions and MSN subtypes deserves further investigation.

Our data do suggest at least a partial selectivity of dependence effects on transmission in the medial striatum, where we observed altered OFC output. Given the similar proportion of OFC inputs onto D1 and D2 SPNs^50^, the lack of changes in OFC transmission onto the indirect pathway is intriguing since the same OFC neuron may send collaterals to both D1 and D2 SPNs. This raises an additional hypothesis that the cell-type specificity of the ethanol-induced decrease in OFC transmission is post-synaptically mediated in a retrograde manner; one potential target being post-synaptic endocannabinoid release and retrograde activation of cannabinoid type-1 receptors located on OFC terminals^21^.

Furthermore, it is unclear how the selective reduction in OFC-D1 SPN transmission would alter direct pathway function and output. Given the convergence of numerous associative cortical inputs onto the same SPN within this DMS region^65^, the relative importance of a decrease in excitatory drive from a portion of inputs on the overall D1 SPNs function and output during action selection is unknown. From previous work, we do know that goal-directed learning differentially alters plasticity of D1 and D2 SPNs in DMS, increasing the AMPA/NMDA ratio in D1 but not D2 SPNs^66^. This suggests that the reduced glutamatergic input from OFC could affect the necessary D1 SPNs plasticity that may sustain goal-directed control. Interestingly, previous work has found that suppression of habitual control is accompanied by decreased output of the direct pathway in DLS^67^, suggesting that direct pathway activity in DLS or DMS is necessary for habitual and goal-directed behavior, respectively. Our finding that CIE results in a selective disruption to corticostriatal circuits in a projection and cell-type-specific manner emphasizes the need for highly specific circuit interrogation in the examination of disordered action selection in disease states.

The present findings highlight the effect drug dependence has on cortical-based decision-making. Although the focus has largely been on bottom up driven transitions underlying the transition from goal-directed to habitual control^2,3,14^, the present findings highlight the contribution of top down processes in this biased use of habitual processes. Alteration of OFC function has frequently been observed in drug dependence^25,29,31,68^, and in particular, ethanol dependence alters OFC function and alcohol-related behaviors^36,69,70^. Reducing OFC activity increased quinine-insensitive alcohol drinking in ethanol-dependent mice^36^, further suggesting that reduced OFC control in dependence escalates habitual-like behaviors. Here we found that increasing the activity of OFC neurons was sufficient to restore goal-directed control and overcome the bias towards habitual action control (Fig. 4). This non-physiological increase in OFC activity was neither temporally nor spatially specific, as we aimed to counter the observed decrease in OFC excitability as well as reduced OFC-DMS transmission. Therefore, the effectiveness of increasing OFC activity on restoring goal-directed control suggests OFC is a viable area to target in the treatment of drug dependence.

## Methods

### Mice

All experiments were approved by the Institutional Animal Care and Use Committees of the University of California San Diego. C57BL/6 J and B6.129S2-Emx1^tm1(cre)Krj^/J (Emx1-Cre) mice^71^ were used for behavioral experiments. B6.FVB(Cg)-Tg(Drd1-cre)EY266Gsat/Mmucd (D1-Cre), B6.FVB(Cg)-Tg(Adora2a-cre)KG139Gsat/Mmucd (A2a-Cre)^72^, and B6.Cg-Tg(Drd1a-tdTomato)6Calak/J (D1-tdTomato)^73^ were used for electrophysiological recordings. All mouse lines were obtained from Jackson Laboratory or Mutant Mouse Resource and Research Center (MMRRC) and bred with C57Bl/6 J mice (Jackson Laboratory) for one generation, in-house. Adult (>8 weeks) male and female mice were housed in groups of one to four, with mouse chow and water ad libitum unless stated otherwise, and were kept on a 14 h light/10 h dark cycle.

### Viral injections

Mice were anesthetized with isoflurane and were given stereotaxically guided injections into the OFC (coordinates from Bregma in mm: anterior [A], 2.70; medial [M] ± 1.65; ventral [V]: 2.65). Emx1-Cre and C57BL6/J mice used for behavioral experiments were injected with 200 nl of AAV5-hSyn-DIO-hM3D-mCherry or AAV5-hSyn-DIO-mCherry in the OFC. D1-Cre, A2a-Cre and D1-tdTomato mice were used for patch-clamp recordings and were coinjected with 100 nl AAV5-CamKIIa-GFP-Cre and 100 nl AAV5-Ef1a-DIO-ChR2-eYFP in the OFC. To identify D1 and D2 SPNs, D1 Cre and A2a Cre were also injected with 200 nl AAV5-hSyn-DIO-mCherry in the DMS ([A], 0.5; [M], ± 1.5; [V], 3.25). Viral spread was assessed by imaging the extent of fluorescence in brain slices (Olympus MVX10).

### Chronic intermittent ethanol exposure and repeated withdrawal

Multiple cohorts of mice were exposed to four rounds of ethanol vapor or air (behavioral experiments cohort *n* *=* 3, ex vivo experiments cohort *n* = 4, and for OFC activation cohort *n* = 3). Each round consisted of 16 h of vapor exposure followed by an 8 h withdrawal, repeated for 4 consecutive days. Ethanol was volatilized by bubbling air through a flask containing 95% ethanol at a rate of 2–3 l/min. The resulting ethanol vapor was combined with a separate air stream to give a total flow rate of approximately 10 l/min, which was delivered to the mice housed in Plexiglas chambers (Plas Labs Inc). Blood ethanol concentrations were collected at the end of each round from sentinel mice (mean BEC = 34.7 ± 2.0 mM). No pyrazole or loading ethanol injections were given prior to placement in vapor chambers.

### Operant training

Training was conducted as previously described^19,21^. Two days prior to training, mice were food restricted and maintained at ~90% of their baseline body weight throughout training and testing. Mice were placed in sound attenuating operant boxes (Med-Associates) and were trained to press a single lever (left or right) for a food reinforcer (chow pellet or 20% sucrose solution). Each mouse was trained in two contexts daily, differentiated by the presence of clear Plexiglas side-walls, or black and white striped plexiglass side-walls. In each context, mice were first trained to retrieve the outcome, in the absence of levers, under a random time schedule (RT60) in which the outcome was delivered on average every 60 s. Mice were then trained on a continuous reinforcement schedule (CRF) in each context in which each lever press produced a single outcome, and the maximum number of reinforcers earned in 3 sessions being 5, 15, and 30, respectively. Following CRF training, mice were trained under a RI schedule in one context and RR schedule in the remaining context. Mice received 2 days of training in RI30 (the first lever press after an average 30 s produces the outcome) and RR10 (on average the 10th lever press produces the outcome), followed by four days under RI60 and RR20. Sessions ended in each context after 15 reinforcers were earned or after 60 min had elapsed. Each day 1–4 h following schedule training, mice had 1 h exposure to a separate outcome (20% sucrose solution or food pellets) in their home cage.

Devaluation testing through sensory-specific satiation was conducted across 2 days: a valued day and a devalued day. Mice were allowed to prefeed for 1 h on the home-cage control outcome (valued day), or the outcome previously earned by lever pressing (devalued day). Mice that did not consume pellets or sucrose during prefeeding were excluded from subsequent analysis. Each day immediately following prefeeding, mice were placed into each context for 5 min where the number of lever presses made were recorded but no outcome was delivered. For groups that received CNO or saline (1 mg/kg, 10 ml/kg), mice were given an intraperitoneal injection 15–30 min prior to prefeeding. Mice in all experimental groups were counterbalanced for schedule exposure, order of devaluation testing, outcome, and lever position. The order of context exposure was kept constant across training and testing. Investigators were not blind to the experimental groups. A subset of Air and CIE mice underwent a post-test feeding assay for 1 h immediately after operant testing on each of the devaluation days.

### Brain slice preparation

Mice were at least 16 weeks of age at the time of slice preparation. Coronal slices (250 μm thick) containing the OFC or DMS were prepared using a Pelco easiSlicer (Ted Pella Inc., Redding, CA). Mice were anesthetized by inhalation of isoflurane, and brains were rapidly removed and placed in 4 °C oxygenated ACSF containing the following (in mM): 210 sucrose, 26.2 NaHCO_3_, 1 NaH_2_PO_4_, 2.5 KCl, 11 dextrose, bubbled with 95 O_2_/5% CO_2_. Slices were transferred to an ACSF solution for incubation containing the following (in mM): 120 NaCl, 25 NaHCO_3_, 1.23 NaH_2_PO_4_, 3.3 KCl, 2.4 MgCl_2_, 1.8 CaCl_2_, 10 dextrose. Slices were continuously bubbled with 95 O_2_/5% CO_2_ at pH 7.4, 32 °C, and were maintained in this solution for at least 60 min prior to recording.

### Patch-clamp electrophysiology

Whole-cell patch-clamp recordings were made in identified D1 and D2 SPNs of the DMS and pyramidal cells of the OFC. In D1-tdTomato mice, D1+ cells were identified as D1 SPNs and D1− cells as D2 SPNs. Cells were identified using an Olympus BX51WI microscope mounted on a vibration isolation table. Prior to patching onto a cell, the presence of td-Tomato expression was used to verify cell-type as well as eYFP expression for terminal expression of ChR2. eYFP expression was never observed in SPN cell bodies. Recordings were made in ACSF containing (in mM): 120 NaCl, 25 NaHCO_3_, 1.23 NaH_2_PO_4_, 3.3 KCl, 0.9 MgCl_2_, 2.0 CaCl_2_, and 10 dextrose, bubbled with 95 O_2_/5% CO_2_. ACSF was continuously perfused at a rate of 2.0 ml/min and maintained at a temperature of 32 °C. Picrotoxin (50 µM) was included in the recording ACSF to block GABA_A_ receptor-mediated synaptic currents. For experiments measuring asynchronous release, Ca^2+^ was replaced with 2 mM Sr^2+^ in the recording solution^54^. Recording electrodes (thin-wall glass, WPI Instruments) were made using a PC-10 puller (Narishige International, Amityville, NY) to yield resistances between 3–6 MΩ. For current clamp experiments, electrodes were filled with (in mM): 135 KMeSO_4_, 12 NaCl, 0.5 EGTA, 10 HEPES, 2 Mg-ATP, 0.3 Tris-GTP, 260–270 mOsm (pH 7.3). For voltage clamp experiments, electrodes were filled with (in nM): 120 CsMeSO_4_, 15 CsCl, 8 NaCl, 10 HEPES, 0.2 EGTA, 10 TEA-Cl, 4 Mg-ATP, 0.3 Na-GTP, 0.1 spermine, and 5 QX-314-Cl. Access resistance was monitored throughout the experiments. Cells in which access resistance varied more than 20% were not included in the analysis.

### Data acquisition

Glutamatergic afferents were stimulated either electrically or optically. For electrical stimulation, a stainless steel bipolar stimulating electrode (FHC, Inc.) was placed dorsal to the recording electrode, about 150–300 μm from the cell body. Optical stimulation was done using 470 nm blue light (4 ms) delivered via field illumination using a high-power LED (LED4D067, Thor Labs). Light intensity was adjusted to produce optically evoked excitatory post-synaptic currents (oEPSCs) with a magnitude of 100–300 pA. Recordings were made using a MultiClamp 700B amplifier (Molecular Devices, Union City, CA), filtered at 2 kHz, digitized at 10 kHz with Instrutech ITC-18 (HEKA Instruments, Bellmore, NY), and displayed and saved using AxographX (Axograph, Sydney, Australia). For PPR, 2 EPSCs were evoked separated by an ISI of 50–250 ms for 5–10 trials, collected at 0.1 Hz. Measurements of frequency and amplitude of asynchronous release in Sr^2+^ containing solution were restricted to 50–2050 ms after stimulus onset for 30 trials, collected at 0.05 Hz. Data from each neuron within a treatment group was combined and presented as mean ± SEM.

### Statistical analysis

Statistical significance was defined as an alpha of *p* < 0.05. To ensure reproducibility of any observed effects, multiple cohorts (at least three cohorts) of Air and CIE exposure were used for all experiments except the experiment looking at asynchronous release (where only two cohorts were used). No effect of cohort was observed. Sample size was determined from previous studies and power analyses on necessary to detect action shifting in control mice. Statistical analysis was performed using GraphPad Prism 6 (GraphPad Software) and JASP. Acquisition data, including lever presses, response rate, rewards earned, and head entries were analyzed using three-way repeated measures ANOVA (day × context × CIE exposure or group). For outcome devaluation testing, three-way repeated measure ANOVAs (context × devaluation state × CIE exposure or group) were performed to examine differences in the pattern of responding. For the outcome devaluation test, lever presses in the valued and devalued states were normalized to total lever pressing (valued + devalued) in each context. In addition, one-sample *t* tests analyses were conducted on the distribution of lever presses to examine whether mice differentially distributed lever presses between valued and devalued states (i.e., did they differ from 0.5 which indicates equal lever presses made between valued and devalued states). We also calculated a devaluation index for each mouse in each context by applying the following equation: [(valued lever presses – devalued lever presses)/(valued lever presses + devalued lever presses)]. We then applied a two-way repeated measures ANOVA (CIE exposure × context) to assess whether there was a within-subject shift in the degree of goal-directed control between contexts, followed by Bonferroni-corrected post hoc follow-ups performed between contexts within a group. We then used a one-sample *t* test against a hypothetical value of 0 (indicating equal pressing between states) to examine the degree to which lever pressing was goal-directed. For patch-clamp experiments, action potential firing and PPR data were analyzed using a two-way ANOVA with Bonferroni-corrected post hoc analyses. Frequency and amplitude of asynchronous release were analyzed using a two-tailed Student’s *t* test. Data are presented as mean ± SEM. No significant differences in the spread of variance were observed between groups, and all data was normally distributed.

### Data availability

All relevant data are available from the authors upon request.

## Electronic supplementary material

Supplementary Material
