# Supplementary material for: Chronic alcohol exposure disrupts top-down control over basal ganglia action selection to produce habits
Source: Nat Commun. 2018 Jan 15;9:211. doi: 10.1038/s41467-017-02615-9 (PMC5768774; doi:10.1038/s41467-017-02615-9)
Supplement: Supplementary file 1 — Supplementary Material [file 41467_2017_2615_MOESM1_ESM.pdf]

Supplementary figures

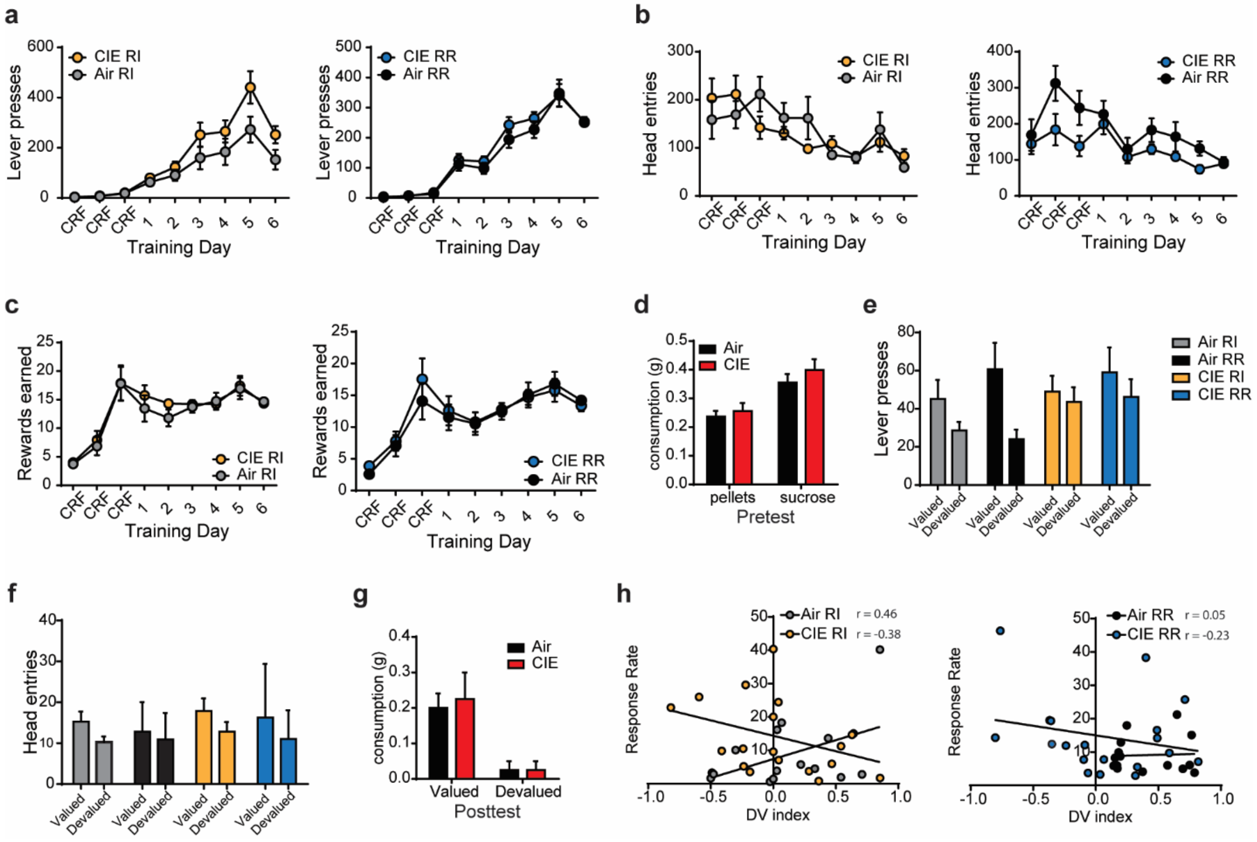

Supplementary Figure 1. Acquisition and performance of lever-pressing behavior and outcome devaluation in Air and CIE mice. (a-c) 3-way repeated measures ANOVA (Day x Context x CIE Exposure) was performed on all acquisition data; for ease of presentation data is presented by context. **(a)** Lever presses made by Air and CIE mice similarly increased across training (no 3-way or 2-way interactions (Fs < 1.70); main effect of day ($F_{8,112}$=68.19, p < 0001); no main effect of Context or CIE Exposure (Fs < 0.18). **(b)** Head entries differentially changed across acquisition for Air and CIE mice. There was a significant 3-way interaction ($F_{8,112}$ = 2.24, p = 0.03), significant two-way (Day x CIE Exposure) interaction ($F_{8,112}$=2.17, p 0.03), and a main effect of Day ($F_{8,112}$=15.71, p < 0.001), but no other significant interactions or main effects (Fs < 1.40). **(c)** Air and CIE mice similarly earned more rewards across training as indicated by a lack of a significant 3-way interaction or any 2-way interactions (Fs < 1.91), but significant main effect of Day ($F_{8,112}$ = 14.15, p < 0.001). No other main effects were observed (Fs < 2,13). **(d)** Consumption was not different between Air and EtOH during the devaluation procedure (unpaired t-test, p > 0.05). **(e)** A repeated measures ANOVA (Context x Devaluation state x CIE Exposure) did not show an interaction or main effect of context (Fs < 1.6), but did reveal a significant context x devaluation state interaction ($F_{(1, 32)}$ = 5.30, p = 0.02) and a trend toward a main effect of devaluation state ($F_{(1, 32)}$ = 4.0, p = 0.05). **(f)** Head entries during devaluation testing were similar in Air and CIE mice (no interactions or main effects (Fs < 1.3). **(g)** Consumption of the outcome was measured immediately after the extinction test in a subset of mice (Air *n* = 4, CIE *n* = 4). There were no differences in posttest consumption between Air and CIE mice (unpaired t-test, p > 0.05). **(h)** Scatter plots of the devaluation index (lever presses valued state−lever presses devalued state)/(lever presses valued state + lever presses

devalued state) in the RI (left) and RR (right) context versus the response rate (lever presses/min) on the last day of training in each context. r = Pearson correlation analysis.

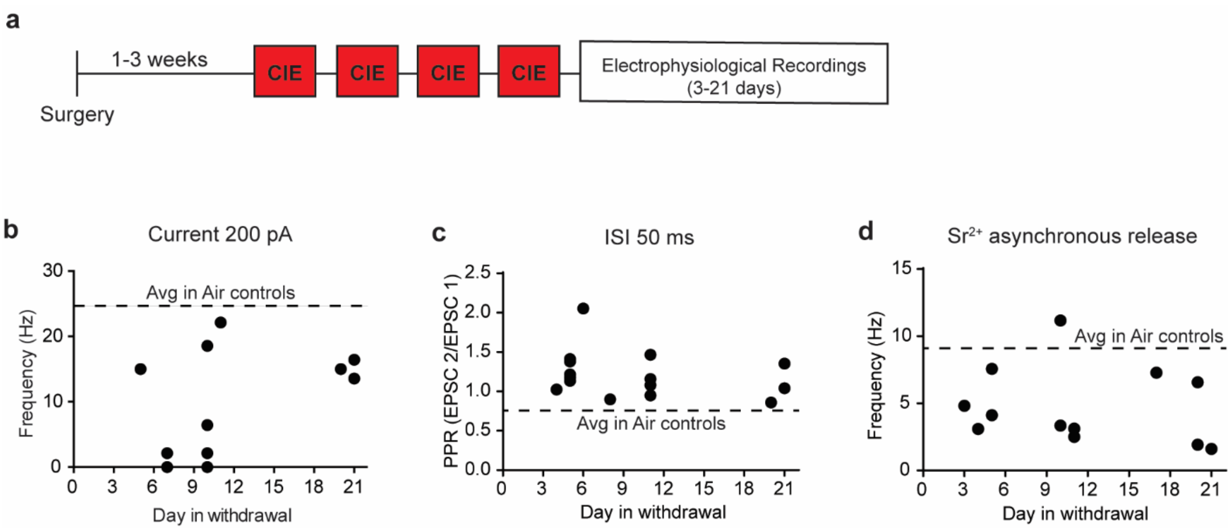

Supplementary Figure 2. CIE induced changes in OFC-DMS transmission across recording days. (a) Experimental timeline for cohorts of mice used for electrophysiological recordings. Mice were injected with AAV ChR2 in the OFC and allowed 1-3 weeks to recover before exposure to the CIE procedure. **(b-d)** Electrophysiological recordings were made 3-21 days in withdrawal. **(b)** The effects of CIE exposure on OFC excitability (Frequency of AP firing at 200 pA), **(c)** PPR in D1 SPNs (interstimulus interval of 50 ms) and **(d)** frequency of asynchronous release onto D1 SPNs were consistent throughout the 21 day withdrawal period. Data points represent individual cells from each group. Dotted line represents the average for Air controls for each group.

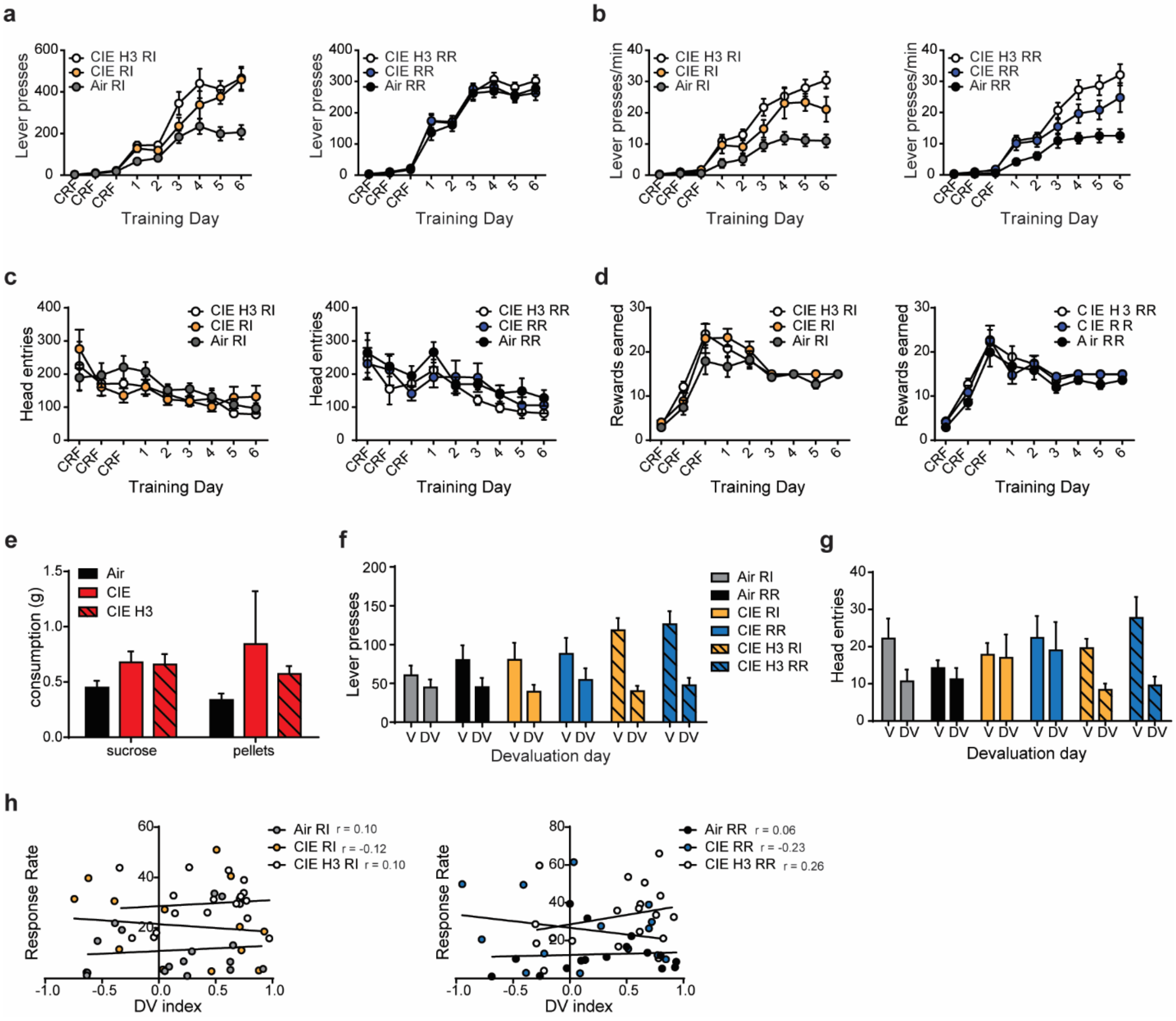

Supplementary Figure 3. Acquisition and performance of lever-press behavior and outcome devaluation in Air, CIE, and CIE H3 mice. 3-way repeated measures ANOVA (Day x Context x CIE Exposure) was performed on all acquisition data; for ease of presentation data is presented by context. **(a)** Treatment groups differentially increased lever pressing in the RI context across training. This was supported by a 3-way ANOVA on lever presses that showed a significant 3-way interaction (Training Day x Context x Group: $F_{16,368} = 3.58$, $p < 0.001$), significant two-way interactions of Context x Group ($F_{2, 46} = 7.60$, $p = 0.001$), Training Day x Group ($F_{16, 368} = 3.43$, $p < 0.001$), Training Day x Context ($F_{8, 368} = 7.73$, $p < 0.001$) and a main effect of Training Day ($F_{8, 368} = 161.53$, $p < 0.001$), but no other interactions or main effects. **(b)** A 3-way ANOVA on response rates (Training Day x Context x Group) did not show a significant interaction (F= 1.11) but did show a significant two-way interaction of Training Day x Group ($F_{16, 368} = 7.40$, $p < 0.001$) and a main effect of Training Day ($F_{8, 368} = 102.26$, $p < 0.001$), but no other interactions or main effects. **(c)** A 3-way ANOVA of head entries did not show a significant interaction (F= 1.10) but did show main effects of Context ($F_{1, 46} = 4.51$, $p = 0.04$) and Training Day ($F_{8, 368} = 7.00$, $p < 0.001$). No other interactions or main effects were observed. **(d)** A 3-way ANOVA of rewards earned during acquisition showed a significant 3-way interaction ($F_{16, 368} = 2.11$, $p = 0.008$) and a main effect of training day ($F_{8, 368} = 51.04$, $p < 0.001$), but no other interactions or main effects. **(e)** No difference in prefeeding consumption of pellets (one-way

ANOVA: $F_{(2, 46)} = 2.037$, $p = 0.14$) or sucrose (one-way ANOVA: $F_{(2, 46)} = 0.99$, $p = 0.38$) during the devaluation procedure. **(f)** A 3-way ANOVA on lever presses during devaluation (Devaluation state x Context x Group) did not reveal a significant 3-way interaction ($F_{(}= 0.761$) but did show a main effect of Devaluation State ($F_{1,46} = 18.50$, $p < 0.001$). No main effect of context or any other significant two-way interactions were observed (Fs' < 2.9). **(g)** A 3-way ANOVA on head entries during devaluation (Devaluation state x Context x Group) did not show a significant 3-way interaction (F= 2.72) but did show a main effect of Devaluation state ($F_{(1, 46)} = 6.58$, $p = 0.01$). **(h)** Scatter plots of the devaluation index (lever presses valued state−lever presses devalued state)/(lever presses valued state + lever presses devalued state) in the RI (left) and RR (right) context versus the response rate (lever presses/min) on the last day of training in each context. r = Pearson correlation analysis.

Supplementary Table 1. Electrophysiological properties of OFC neurons of Air and CIE exposed mice

Group	RMP (mV)	Input Resistance (MΩ)	First spike latency (ms)	Threshold (mV)	AHP (mV)	Amplitude (mV)	Rise time (ms)	Half width (ms)
**Air**	-67.37 ± 0.83	106.33 ± 16.71	131.49 ± 26.40	-36.43 ± 2.18	15.75 ± 1.21	61.37 ± 3.90	0.58 ± 0.09	0.88 ± 0.05
**CIE**	-70.69 ± 1.19*	135.88 ± 20.07	180.49 ± 54.15	-33.65 ± 1.69	16.39 ± 1.94	60.99 ± 4.62	0.59 ± 0.05	0.99 ± 0.06

Avg ± SEM. * $p < 0.05$
